# Multicast Traffic Throughput Maximization through Joint Dynamic Modulation and Coding Schemes Assignment, and Transmission Power Control in Wireless Sensor Networks†

**DOI:** 10.3390/s21103411

**Published:** 2021-05-13

**Authors:** Bartłomiej Ostrowski, Michał Pióro, Artur Tomaszewski

**Affiliations:** Institute of Telecommunications, Warsaw University of Technology, 00-665 Warsaw, Poland; m.pioro@tele.pw.edu.pl (M.P.); a.tomaszewski@tele.pw.edu.pl (A.T.)

**Keywords:** wireless sensor networks, multicast traffic, throughput maximization, transmission scheduling, TDMA, transmission power control, IoT, MCS, mixed-integer programming

## Abstract

The paper concerns multicast packet traffic throughput maximization in multi-hop wireless sensor networks with time division multiple access to radio channel. We assume that the modulation and coding schemes (MCSs) that are used by the (broadcasting) nodes as well as the transmission power of the nodes are adjustable. This leads to the main research question studied in this paper: *to what extent traffic throughput can be increased by proper MCSs assignment and transmission power control (TPC) at the nodes?* To answer this question, we introduce mixed-integer programming formulations for joint MCSs assignment and TPC optimization, together with a solution algorithm. Finally, we present a numerical study illustrating the considerations of the paper. The numerical results show a significant gain being achieved by proper MCSs assignment, which is further increased by applying TPC.

## 1. Introduction

Wireless sensors networks (WSNs) are an important element of modern networking, mainly due to the rapidly growing range of their applications [1], including the military, smart cities, environment, health care, and agriculture [2]. Some interesting recent applications examples of WSNs in transportation system can be found in [3], while [4,5] discuss the role of WSNs in COVID-19 detection and monitoring. WSNs are often composed of the numerous low-cost nodes, thus they are usually perceived in the context of the limited resources when it comes to power, memory, and computational capabilities. However, although initially envisioned to serve simple services, such as environmental data monitoring, they have recently become one of the most promising solution for Internet of Things (IoT). Therefore, they are not only the limited resources that should be taken into account while designing a solution for WSNs, but also some Quality of Service (QoS) requirements, such as bandwidth or delay [6]. Particularly, such requirements are especially important in Industrial Internet of Things where a huge amount of data requires a high network capacity to avoid severe network congestions [7] (for a comprehensive study of the problem of designing a high-performance wireless network, i.e., the networks with Gbps data rates and 10-µs-level cycle time, in industrial environment the reader is referred to [8], and for the recent discussion on IoT in general the reader is referred to [9,10]). However, in the existing literature, this fact is often neglected and the main focus is on the energy efficiency or the network lifetime maximization, and not on the performance requirements to be fulfilled by the network. Thus, we believe that the traffic throughput maximization problem is of a great importance in the context of WSNs.

As far as the traffic throughput maximization problem is concerned, it is important to take multicast transmission into account. Note that, in the wireless networks multiple nodes can receive the packets broadcast in the same transmission, as long as they are within transmitter’s transmission range (clearly, it is not possible in the wired networks where multicast transmission requires a copy of a given packet to be sent to each recipient). Multicast transmission can lead to a significant gain of throughput as well as to the lower energy consumption because of the broadcast nature of wireless medium. Obviously, this statement is well justified in the existing literature. For example, paper [11] discusses the advantageous of multicast transmission in WSNs, while algorithms and protocols for multicast transmission in WSN can be found, for example, in [12,13]. Moreover, some papers, such as  [14,15,16], consider multicast transmission for the particular throughput maximization problem that we dealt with in this paper. In particular, the numerical section presented in [16] contains a comparison of unicast vs. multicast routing efficiency, highlighting the gain that is achieved by utilizing multicast in WSNs.

Two other factors that play a crucial role in traffic throughput maximization are proper modulation and coding schemes (MCSs) assignment as well as the transmission power control (TPC). Because of the tradeoff between the number of simultaneous transmissions and transmission data rates the selection of MCSs is not at all straightforward; however, it leads to a significant gain of traffic throughput, as shown in [17]. Such a gain can be further increased by applying TPC (for example, using the lowest power sufficient to realize a transmission can potentially result in the increase of the number of parallel transmissions). Unfortunately, joint MCSs assignment and TPC turns out to be a very complex task.

At this point, it is important to notice that, although MCSs assignment and TPC can be optimized separately (e.g., by optimizing MCSs assignment for a given and fixed transmission power level, and then, on top of that, optimizing TPC), such an approach will, in general, not lead to optimal solutions. This is because the transmission power level used in the first step of such a procedure can exclude some MCSs from feasible solutions. On one hand, a low transmission power level can exclude the MCSs with high transmission data rates and relatively low transmission range and, on the other hand, the same MCSs can be excluded by a high transmission power level because of the high interference level when other nodes transmits concurrently (note that MCSs with high transmission date rates require strong signal to be properly decoded). Therefore, it is crucial to treat the optimization of MCSs assignment and TPC jointly.

In the light of the foregoing, in this paper we study the following research question: *to what extent traffic throughput can be increased by proper modulation and coding schemes assignment and transmission power control at the nodes?* To answer this question, we provide the optimization model for multicast traffic throughput maximization in WSNs utilizing time division multiple access (TDMA) to radio channel, where both MCSs and the transmission power of the broadcasting nodes are adjustable. We assume that the MCSs as well as the transmission power used by the broadcasting nodes can be switched from a time slot to a time slot. Two TPC cases are considered in the paper: discrete TPC and continuous TPC. In the former, the transceivers can use only a discrete, usually small, set of power levels (this is common when it comes to low-cost sensor nodes), while, in the latter, the transmissions power applied by the nodes can change continuously. The presented optimization model is the extension of the model that was introduced in [14] (the model was further extended in [17] to include dynamic MCSs assignment) that make it possible to deal with the complex problem of including transmission power control into throughput optimization in WSNs with dynamic MCSs assignment that serve multicast traffic. The provided optimization model allows us to treat the considered problem in a rigid mathematical way, thus it can be used as a benchmark while designing algorithms and protocols to be implemented in the real networks. To the best of our knowledge, such a model does not exist in the literature and, together with the numerical study that illustrates the considerations of the paper, constitute the main contributions of this work.

As written above, we consider TDMA-based WSNs. Note that using TDMA is well justified in the given context, since it outperforms other medium access control schemes in terms of traffic throughput. It is also worth mentioning that TDMA is considered to be one of the most promising solutions for energy efficient WSNs [18]. The list of radio technologies supporting TDMA includes WirelessHART, ISA100, and the family of IEEE 802.15.4-based low-rate personal area networks (LR-WPAN).

The rest of this paper is organized as follows. In Section 2, we discuss the related work on multicast transmission, MCSs assignment, and TPC in WSNs. Section 3 presents the network description and notations used in this paper. Problem statement and the corresponding mixed-integer programming (MIP) formulations, together with a solution algorithm, are presented in Section 4, Section 5 and Section 6. Subsequently, numerical results illustrating considerations of this paper are discussed in Section 7. Finally, Section 9 concludes the paper.

## 2. Related Work

Wireless sensors networks are widely discussed in the literature. In particular, a lot of papers concern the specific aspects studied in this paper, i.e., multicast transmission, modulation and coding schemes assignment, and transmission power control in WSNs.

A majority of papers on multicast transmission in WSNs is devoted to the problem of finding an energy efficient routing, usually by means of some heuristic algorithms. Ref. [19] presents a heuristic distributed minimum transmission multicast routing protocol for WSNs, Ref. [20] studies an energy efficient multicast geographic routing, paper [13] proposes a multipath scheme applied on a multicast-based hierarchical routing that gives better energy utilization, which leads to the network lifetime improvement, and [21] makes use of a genetic algorithm for solving the Network Coding Resource Minimization problem. Another important group of papers concerning multicast transmission in WSNs contains the papers focusing on delay minimization. Ref. [22] proposes both centralized and distributed algorithms for delay-bounded scheduling in duty-cycled WSNs, Ref. [23] focuses on the latency and network lifetime tradeoff, and [24] deals with an energy efficient, minimum-delay flooding in duty-cycled WSNs.

Additionally, the problem of modulation and coding schemes assignment has been to a certain extent studied in the literature. Some interesting conclusions can be found in [25], where the authors pointed out that using more robust MCSs instead of these with the highest data rates can lead to the improvement of the overall network throughput. Ref. [26] studies channel coding for high performance wireless control; particularly, the authors present a comprehensive analysis of the packet coding schemes used in industrial WSNs and propose some rules for choosing the most promising coding schemes. Finally, Ref. [27] provides the performance analysis of the efficient coding schemes for WSNs assuming BPSK modulation and a Gaussian channel.

Because transmission power control is one of the most important issues when it comes to WSNs and IoT, it is studied in many papers. For example, paper [27] proposes a game-theoretic power control mechanism for WSNs with imperfect information. Next, paper [28] presents plenty of interesting engineering takeaways on wireless mesh networks (WMNs) configuration, including power control, which result in throughput maximization. Finally, Ref. [29] studies the effects of TPC on the energy consumption of WSNs. A distinguishable and interesting group of work dealing with TPC in WSNs is composed of the articles that make use of machine learning. Paper [6] takes advantage of reinforcement learning to provide a distributed solution for choosing the lowest transmission power possible and, thus, to minimize both energy consumption and interference. Additionally, paper [30] utilizes reinforcement learning to deal with the average throughput maximization per total consumed energy in WSNs in both point-to-point and multinodes scenarios. As far as the recent research is concerned, the following items should be mentioned. Paper [31] deals with energy-aware dynamic allocation problem in the context of Social Internet of Vehicles, Ref. [32] studies the problem of power control and unmanned aerial vehicle deployment for IoT networks, while [33] proposes a novel self-adaptive power control-based enhanced energy-aware approach to reduce the energy consumption and enhance the battery lifetime and reliability.

Despite a vast amount of the literature concerning WSNs, to the best of our knowledge, the papers on throughput maximization as well as mathematical modelling are not common—most of the existing work treats the problem from the viewpoint of engineering, without an attempt to provide a rigid mathematical model. Although some interesting results in this area can be found in [34,35], they are limited to unicast traffic. In [34], mixed-integer programming formulations for max-min fair flow optimization in WMNs including both static and dynamic MCSs assignments, while [35] provides a complete optimization model for joint routing, power control, scheduling, channel assignment as well as rate control optimization in WMNs. Therefore, we believe that the work presented in this paper, which is an extension of the model that was introduced in [14] (extended in [17]), is the first complete optimization model for traffic throughput maximization admitting dynamic MCSs assignment and TPC in WSNs for multicast traffic.

## 3. Network Description

In this section, we present the basic assumptions of our optimization model together with the notations that are summarized in Table 1.

The considered WSN is modeled by means of a directed graph G=(V,A), where V is the set of nodes and A⊆V×V∖{(v,v):v∈V} is the set of directed links (arcs). The beginning of arc *a* will be denoted by b(a), while its end by e(a). We assume that (w,v)∈A if, and only if, node *v* is in the transmission range of node *w*, i.e., when the signal-to-noise-ratio (SNR) condition is satisfied at node *v* when node *w* is transmitting and no other node is transmitting at the same time. Such a condition can be formally expressed by the following formula: $\frac{p(w,v)}{\eta }\geq \gamma \left(m\right)$, where p(w,v) is the power of signal transmitted by node *w* and received by node *v*, η is the noise power, m∈M is a modulation and coding scheme (MCS) used by node *w* (M={1,2,…,M} denotes the set of available MCSs), and γ(m)>1 is the assumed power ratio threshold for a given MCS *m*. Besides the power ratio threshold γ(m), each MCS *m* is also characterized by the transmission rate B(m) that is expressed in Mbps. Note that, because MCSs have different power ratio threshold, the transmission range of a given node depends on a MCS used by this node. For a more detailed description of the radio parameters, the reader is referred to Section 7.1. The set of neighboring nodes connected to *v* by the arcs outgoing from *v* is denoted by $\delta^{+}\left(v\right):=\left\{w\in V,\left(v,w\right)\in A\right\}$. Similarly, the set of neighboring nodes connected by the arcs incoming to *v* is denoted by $\delta^{-}\left(v\right):=\left\{w\in V,\left(w,v\right)\in A\right\}.$

We assume that the network nodes are divided into three pairwise disjoint subsets: sensors S, destinations D, and purely transit nodes Q. Each sensor s∈S is the source of packets (for example, containing measurement data that the sensor collect from the environment) that are to be delivered to multiple destinations specified by the set of destinations D(s)⊆D. We assume that the sensors are capable of both generating and transiting packets, while the destination nodes only terminate traffic, i.e., they are not able to transit packets; clearly, the transit nodes do not generate or terminate packets, and only transit them.

The packets that are generated by sensor *s* are transmitted to their destinations along paths of a multicast tree B(s)=(V(s),A(s)), where V(s)⊆V is the set of its nodes while A(s)⊆A is the set of its arcs (|A(s)|=|V(s)|−1). Clearly, such a tree is routed at *s* and D(s) are its leaves. Note that, at each node *v* of the multicast tree B(s) (except for the leaves), the packets are, in general, transferred to more than one neighboring node, and this is the place when we take advantage of the broadcast nature of radio transmissions, since the packet broadcast from a node *v* reaches all of the nodes within its transmission range of the node *v* anyway.

We consider TDMA-based WSNs, which means that the packets are transmitted within equally long time slots that are grouped into consecutive TDMA frames of *T* time slots each. We assume that each sensor sends out the measurement data at the beginning of each consecutive frame. The frames are repeated periodically and, hence, the transmission patterns that are applied in the consecutive frames are identical, i.e., the set of broadcasting nodes as well as the set of receiving nodes in *t*th slot (t=1,2,…,T) of each frame are the same. A pair composed of the set of (simultaneously) broadcasting nodes and the sets of simultaneously receiving nodes defined for each broadcasting node will be called compatible set (c-set in short) and they will be denoted by *c*. In the following, $\hat{C}$ will denote the family of all c-sets for a given network (note that the size of this family grows exponentially with the network size), and C a selected subfamily of $\hat{C}$. The set of nodes broadcasting in c-set *c* will be denoted by W(c), m(c,w),w∈W(c) denotes MCSs assignment, and the set of nodes receiving from node w∈W(c) in c-set *c* will be denoted by U(c,w). Note that any c-set *c* is valid if, and only if, each of the nodes in U(c,w) can decode the signal from *w*, i.e., the nodes receiving from *w* are not interfered by the signals that are simultaneously broadcast from the nodes in W∖{w}. Such a condition can be expressed in the following formula:

(1) $$\frac{p(w,u)}{\eta +\sum_{v\in W\setminus \{w\}}p\left(v,u\right)}\geq \gamma \left(m\left(c,w\right)\right),\;w\in W,u\in U\left(c,w\right).$$

The above c-set description can by formally expressed by the set of linear inequalities imposed on the variables that characterize the sets W(c) and U(c,w), MCS assignment m(c,w),w∈W(c), and the transmission power of each node; such a formal definition will be introduced in the following part of the article.

## 4. Problem Formulation

As already mentioned, the paper deals with packet throughput maximization, which in the considered network setting is equivalent to the problem of finding a TDMA frame of minimum size such that the network is capable to deliver all packets generated by sensors to all their destinations with a finite delay. This is because the packets are generated at the beginning of each consecutive frame, thus the shorter the frame the greater the packets arrival intensity. In the paper we will maximize traffic throughput by means of this equivalence.

In order to formally formulate the above characterized optimization problem we use a mixed-integer programming formulation using the following sets of variables:

- $h_{swc},s\in S,w\in V,c\in C$, non-negative continuous, expresses the amount od data from stream *s* to be broadcast from node *w* when c-set *c* is applied,
- $T_{c},c\in C$, integer, denotes the number of slots in the frame in which c-set *c* is active.

In order to ensure stable delivery (i.e., with a finite delay) of the packets to their destinations, the following constraints are imposed:

(2a) $$\begin{array}{cc} \; & \sum_{c\in C(a)}h_{sb(a)c}\geq n\left(s\right)y\left(s,a\right),\;s\in S,a\in A \end{array}$$

(2b) $$\begin{array}{cc} \; & \sum_{s\in S}h_{swc}\leq B\left(m\left(c,w\right)\right)T_{c},\;c\in C,w\in W\left(c\right), \end{array}$$

where C(a) denotes the set of c-sets in C containing arc *a*, n(s) denotes data volume (expressed in Mb) generated at *s* at the beginning of each consecutive frame, and y(s,a) is a given parameter equals 1 if, and only if, arc *a* belongs to the routing tree B(s). Constraint (2a) ensures that data from a given stream will be transmitted over all arcs belonging to its routing trees within a single frame, while constraint (2b) ensures that the number of slots $T_{c}$ is sufficient to realize all scheduled broadcasts.

Note that the above constraints assume fixed (predefined) routing trees. However, if TPC and dynamic MCSs assignment are to be embedded in the frame minimization problem it is essential to optimize the routing trees as well (since adjusting the transmitting power and MCS used by a node influences the signal range, and in consequence the predefined routing trees can easily exclude some power levels or MCSs from the feasible solutions). This can be achieved by treating the parameters y(s,a) as binary variables (denoted by $y_{sa}$) and adding flow variables $z_{swa},s\in S,w\in D\left(s\right),a\in A$. The flow variables corresponding to a given sensor s∈S specify the paths from *s* to its destinations in D(s): the set of arcs forming the selected path from *s* to w∈D(s) is determined as $\left\{a\in A:z_{swa}=1\right\}$. Certainly, the flow variables depend on variables $y_{sa},a\in A,$ that define the routing tree B(s) (with $A\left(s\right)=\left\{a\in A:y_{sa}=1\right\})$. The final form of the frame minimization problem (FMP), formulated by adding additional constraints imposed on the new variables and an objective function is as follows:

(3a) $$\begin{array}{cc} \; & FMP(C):\;\min\;T=\sum_{c\in C}T_{c} \end{array}$$

(3b) $$\begin{array}{cc} \; & \sum_{c\in C(a)}h_{sb(a)c}\geq n\left(s\right)y_{sa},\;s\in S,a\in A \end{array}$$

(3c) $$\begin{array}{cc} \; & \sum_{s\in S}h_{swc}\leq B\left(m\left(c,w\right)\right)T_{c},\;c\in C,w\in W\left(c\right) \end{array}$$

(3d) $$\begin{array}{cc} \; & \sum_{a\in \delta^{-}\left(v\right)}z_{swa}+I\left(s,w,v\right)=\sum_{a\in \delta^{+}\left(v\right)}z_{swa},\\ \; & \;\;\;\;\;\;\;\;s\in S,w\in D(s),v\in V \end{array}$$

(3e) $$\begin{array}{cc} \; & z_{swa}\leq y_{sa},\;s\in S,w\in D\left(s\right),a\in A \end{array}$$

(3f) $$\begin{array}{cc} \; & y_{sa}\in B,\;s\in S,a\in A \end{array}$$

(3g) $$\begin{array}{cc} \; & z_{swa}\in R_{+},\;s\in S,w\in D\left(s\right),a\in A \end{array}$$

(3h) $$\begin{array}{cc} \; & h_{swc}\in R_{+},\;s\in S,c\in C,w\in W\left(c\right);\;\;T_{c}\in R,\;c\in C. \end{array}$$

Constraints (3d) and (3e) are the new constraints that ensure correctness of the constructed routing trees (indicator I(s,w,v) used in (3d) equals 1 for v=s, and −1 for v=w). Finally, the objective function (3a) minimizes the total numbers of time slots used by the frame, i.e., it minimizes the sum of the numbers of time slots assigned to the c-sets actually used in the frame. The so described formulation was originally presented in [17]. It should also be emphasized that the frame minimization problem represented by the above formulation is NP-hard, since, its basic version is already NP-hard [14].

Note that the above formulation provides an optimal solution only for a given family of c-sets C (and not for the whole c-sets family), and it does not consider power control. Both of these aspects will be addressed during solution process.

## 5. Price-and-Branch FMP Solution Algorithm

In this section we describe the algorithm we propose to solve the problem considered in the article and provide a complexity analysis of the algorithm.

### 5.1. Algorithm Description

As already mentioned, the size of family $\hat{C}$, i.e., the number of all valid c-sets, increases exponentially with respect to the network size. That is why the formulation of FMP(C) is non-compact, with the number of variables and constraints growing exponentially with the network size. Thus, to find a globally optimal solution of FMP, i.e., when all c-sets in $\hat{C}$ are considered, we need a way for generating the c-sets needed for achieving such a solution. Our approach is to apply the column and constraint generation method to the liner relaxation of the MIP formulation (3) of FMP(C). Such a linear relaxation is obtained from formulation (3) by relaxing variables $y_{sa}$ and $T_{c}$, i.e, by letting them to be continuous. (Note that in this relaxation constraint $y_{sa}\leq 1$ has not be added since in optimal solutions this condition will be fulfilled.) The problem dual to the considered relaxation is as follows.

(4a) $$\begin{array}{cc} \; & \mathrm{DFMP}(C):\;\max\;W=\sum_{s\in S}\sum_{w\in D(s)}\left(\varphi_{sws}-\varphi_{sww}\right) \end{array}$$

(4b) $$\begin{array}{cc} \; & \sum_{w\in D(s)}\sigma_{swa}\leq n\left(s\right)\lambda_{sa},\;s\in S,a\in A \end{array}$$

(4c) $$\begin{array}{cc} \; & \varphi_{swb(a)}-\varphi_{swe(a)}\leq \sigma_{swa},\;s\in S,w\in D\left(s\right),a\in A \end{array}$$

(4d) $$\begin{array}{cc} \; & \sum_{w\in W(c)}B\left(m\left(c,w\right)\right)\pi_{cw}=1,\;c\in C \end{array}$$

(4e) $$\begin{array}{cc} \; & \sum_{v\in U(c,w)}\lambda_{s(w,v)}\leq \pi_{cw},\;s\in S,c\in C,w\in W\left(c\right) \end{array}$$

(4f) $$\begin{array}{cc} \; & \varphi_{swv}\in R,\;s\in S,w\in D\left(s\right),v\in V \end{array}$$

(4g) $$\begin{array}{cc} \; & \sigma_{swa}\in R_{+},\;s\in S,w\in D\left(s\right),a\in A \end{array}$$

(4h) $$\begin{array}{cc} \; & \lambda_{sa}\in R_{+},\;s\in S,a\in A \end{array}$$

(4i) $$\begin{array}{cc} \; & \pi_{cw}\in R_{+},\;c\in C,w\in W\left(c\right), \end{array}$$

where the dual variables $\varphi_{swv},\sigma_{swa},\lambda_{sa},\pi_{cw}$ correspond, respectively, to the primal constraints (3b)–(3e).

At this point we note that, as it commonly happens in the case of dual problems, DFMP and in particular its objective function (4a), can hardly be interpreted in a rigid but understandable way. Thus, although some helpful intuition could be developed, it is virtually impossible to explain the interpretation of DFMP in a compact manner.

Suppose that $\lambda ={\left(\lambda_{sa}^{*}\right)}_{s\in S,a\in A}$ is a vector of optimal values of dual variables $\lambda_{sa}$ and consider the following expression:

(5) $$P\left(c,\lambda^{*}\right):=\min_{\pi \geq 0,\;\sum_{w\in W(c)}B\left(m\left(c,w\right)\right)\pi_{w}=1}Q\left(\pi ;c\right)$$

where $\pi ={\left(\pi_{w}\right)}_{w\in W(c)}$ and

(6) $$\begin{array}{cc} \; & Q(\pi ;c):=\\ \; & \;\;\sum_{w\in W(c)}\sum_{s\in S}\max\left\{0,\sum_{u\in U(c,w)}\lambda_{s(w,u)}^{*}-\pi_{w}\right\}. \end{array}$$

Now consider the (dual) polytope of DFMP(C∪{c}), i.e., the domain of DFMP(C∪{c}), for a given c-set $c\in \hat{C}\setminus C$. Observe that P(c) expresses the sum of violation of the constraints (4e) corresponding to *c* in this polytope by the vector $\lambda^{*}$ which is optimal for DFMP(C). (Note that the rest of the constraints are not violated in the new polytope.)

Finally, let $c^{*}\in \hat{C}\setminus C$ be a c-set that solves the so called pricing problem (PP):

PP($\lambda^{*}$): Find a c-set *c* in $\hat{C}\setminus C$ that maximizes the value of $P(c,\lambda^{*})$ defined in (5).

Note that when $P(c^{*},\lambda^{*})=0$ then no constraint is violated in DFMP($C\cup \{c^{*}\}$) and optimal solutions of DFMP(C) are also optimal for DFMP($\hat{C}$), and the same holds for the considered linear relaxation of FMP(C). Otherwise, adding $c^{*}$ to DFMP, i.e., to C, will in general decrease the maximum of *W* in (4a) because the dual polytope of DFMP($C\cup \{c^{*}\}$) is a proper subset of the polytope of DFMP(C) (because those solutions of the latter problem that contain $\lambda^{*}$ do not belong to the polytope of DFMP($\hat{C}))$. Thus, since for a given C, the optimum $W^{*}$ of the dual is equal to the optimum $T^{*}$ of the linear relaxation of FMP, adding $c^{*}$ to FMP will in general decrease the optimum of FMP.

To summarize, the following c-set generation algorithm can be used in order to solve the linear relaxation of FMP for the full list of c-sets. 

**CGA**: **c-set generation algorithm**  
**Step-1:**  Define an initial feasible list C of c-sets.  
**Step-2:**  Solve the dual master problem DMP(C) using an LP solver to obtain optimal dual variables $\lambda^{*}$.  
**Step-3:**  Solve the pricing problem PP($\lambda^{*}$) using a MIP solver. If the maximum of the objective function $P^{*}=P\left(c^{*},\lambda^{*}\right)$ is strictly greater than 0, then add the resulting c-set $c^{*}$ to the c-set list C ($C:=C\cup \{c^{*}\}$) and go to Step-2.
**Step-4:**  Otherwise stop: the resulting c-set list $C^{*}$ is sufficient to solve the linear relaxation of FMP to global optimality.    

For completeness, Algorithm 1 shows the pseudocode of CGA, where the following functions are used:

- define_initial_feasible_cSets_list()—the function that generates and returns an initial feasible list C of c-sets
- solve_DMP(C)—the function that solves the dual master problem DMP(C) and returns optimal dual variables $\lambda^{*}$
- solve_PP(C)—the function that solves the pricing problem PP($\lambda^{*}$) and returns a c-set $c^{*}$

**Algorithm 1:** CGA: c-set generation algorithm1:**procedure**CGA2:    C:=define_initial_feasible_cSets_list();3:    new_cSet_generated:=true;4:    **while** (new_cSet_generated=true) **do**5:        $\lambda^{*}:=solve\_DMP\left(C\right)$;6:        $c^{*}:=solve\_PP\left(\lambda^{*}\right)$;7:        **if** $P(c^{*},\lambda^{*})>0$ **then**8:           $C:=C\cup \{c^{*}\}$;9:        **else**10:           new_cSet_generated:=false;11:        **end if**12:    **end while**13:**end procedure**

A simple feasible initial c-set list (i.e., a list C for which FMP(C) is feasible) that can be used for Step-1 is the family of all c-sets with only one node broadcasting to all its neighbors, with a fixed transmission power level.

Finally, for solving the main problem, we use the so called price-and-branch method (see [36]), i.e., we first apply CGA and then solve the basic MIP formulation (3) for the family $C^{*}$ obtained from CGA. For that, we apply a MIP solver.

### 5.2. Algorithm Complexity

Recall that the algorithm we proposed consists of two phases:

**Phase 1**: solving the linear relaxation of FMP.
**Phase 2**: solving the MIP version of FMP with the c-sets generated in the first phase.

In the first phase, we consider the linear relaxation of FMP and, since its formulation is non-compact, we have to solve it by means of the column and constraint generation technique, i.e., we generate the c-sets that are essential to reach optimality of the linear relaxation of FMP. Because the number of all c-sets grows exponentially with the network size, we cannot guarantee that the number of c-sets generated during the process grows polynomially, and not exponentially. Thus, in practice, the number of steps in this phase can grow exponentially with the network size. In each such step, we solve the dual master problem DMP and the pricing problem PP. We solve DMP by means of the simplex algorithm, which is very effective. PP is an NP-hard MIP problem [14] that is solved with branch-and-bound (B&B) algorithm whose complexity is exponential [37].

In the second phase of the algorithm, we use the B&B algorithm to solve the MIP version of FMP for the c-sets that are generated in the first phase (recall that, similarly to PP, this problem is also NP-hard). Thus, the complexity of this phase is also exponential.

However, although the complexity of the proposed algorithm is exponential, the algorithm is capable of solving, as illustrated in Section 7, medium sized problem instances in a reasonable time.

## 6. Pricing Problems

In this section, we present MIP formulations of the pricing problem (PP) for three different cases: no power control, discrete power control, and continuous power control. These formulations are used in Step-3 of CGA. Note that the pricing problem for the case with no power control was already presented in [17]; yet, we present this version of PP below, because it was used in the numerical study for comparison purposes. The derivation of PP without power control can be found in [17], and derivations of the two remaining cases are analogous.

### 6.1. No Power Control

In the case when the network nodes transmit with a fixed and predefined transmission power level, the appropriate PP is as follows:

(7a) $$\begin{array}{cc} \; & \mathrm{PP}(\lambda^{*}):\;\max\;P=-f+\sum_{s\in S}\sum_{w\in V}\sum_{v\in \delta^{+}\left(w\right)}\lambda_{s(w,v)}^{*}U_{swv} \end{array}$$

(7b) $$\begin{array}{cc} \; & X_{w}\geq Y_{wu},\;w\in V,u\in \delta^{+}\left(w\right) \end{array}$$

(7c) $$\begin{array}{cc} \; & X_{w}\leq \sum_{u\in \delta^{+}\left(w\right)}Y_{wu},\;w\in V \end{array}$$

(7d) $$\begin{array}{cc} \; & X_{w}+\sum_{u\in \delta^{-}\left(w\right)}Y_{uw}\leq 1,\;w\in V \end{array}$$

(7e) $$\begin{array}{cc} \; & \sum_{m\in M}z_{w}^{m}=X_{w},\;w\in V \end{array}$$

(7f) $$\begin{array}{cc} \; & p\left(w,u\right)+M\left(w,u,m\right)\left(1-Y_{wu}\right)\geq \\ \; & \;\;\gamma \left(m\right)z_{w}^{m}\eta +\gamma \left(m\right)\sum_{v\in V\setminus \{w,u\}}p\left(v,u\right)Z_{wv}^{m}),w\in V,u\in \delta^{+}\left(w\right),m\in M \end{array}$$

(7g) $$\begin{array}{cc} \; & Z_{wv}^{m}\leq z_{w}^{m},\;\;Z_{wv}^{m}\leq X_{v},\;\;Z_{wv}^{m}\geq z_{w}^{m}+X_{v}-1,m\in M,w\in V,v\in V\setminus \left\{w\right\} \end{array}$$

(7h) $$\begin{array}{cc} \; & U_{swv}\leq g_{sw},\;U_{swv}\leq Y_{wv},\;\;U_{swv}\geq g_{sw}+Y_{wv}-1,w\in V,s\in S,v\in \delta^{+}\left(w\right) \end{array}$$

(7i) $$\begin{array}{cc} \; & \sum_{s\in S}g_{sw}\leq \sum_{m\in M}B\left(m\right)F_{w}^{m},\;w\in V;\;g_{sw}\leq 1,\;s\in S,w\in V \end{array}$$

(7j) $$\begin{array}{cc} \; & F_{w}^{m}\leq \left|S\right|z_{w}^{m},\;\;F_{w}^{m}\leq f,\;\;F_{w}^{m}\geq f-\left|S\right|\left(1-z_{w}^{m}\right),m\in M,w\in V. \end{array}$$

(7k) $$\begin{array}{cc} \; & Y_{wu}\in B,\;w\in V,u\in \delta^{+}\left(w\right);\;X_{w}\in B,\;w\in V \end{array}$$

(7l) $$\begin{array}{cc} \; & z_{w}^{m}\in B,\;w\in V,m\in M;\;Z_{wv}^{m}\in B,\;w\in V,V\in V\setminus \left\{w\right\},m\in M \end{array}$$

(7m) $$\begin{array}{cc} \; & f\in R;\;F_{w}^{m}\in R,\;m\in M,w\in V;\;g_{sw}\in R_{+},\;s\in S,w\in V. \end{array}$$

Note that inequalities (7b)–(7f) define a valid c-set. Constraints (7b) and (7c) force the node to broadcast ($X_{w}=1$) if its broadcast signal is to be received by any of its neighbors ($Y_{wu}=1$); otherwise, $X_{w}$ is forced to be zero. Next, constraint (7d) ensures that, if a node is transmitting, then it cannot receive, and if a node is not transmitting, then it can be receiving, but from, at most, only one neighboring node. Constraint (7e) assures that only one MCS can be used ($z_{w}^{m}=1$) by each broadcasting node. Finally, constraint (7f) expresses the signal-to-interference-to-noise ratio (SINR) condition. The second term of the left-hand side of (7f) is added to cancel the constraint whenever $Y_{wu}=0$ using a "big M” constant i.e., the upper bound on the right-hand side of (7f).

### 6.2. Discrete Power Control

In the discrete power control case, we assume that, in each time slot, each network node can transmit with the transmission power level from a discrete set of available power levels that are denoted by P. Because the transmission power of each node is defined per time slot, we can embed the problem of power assignment into PP, which, in this case, takes the following form:

(8a) $$\begin{array}{cc} \; & max\;\;P=-f+\sum_{s\in S}\sum_{w\in V}\sum_{v\in \delta^{+}\left(w\right)}\lambda_{s(w,v)}^{*}U_{swv} \end{array}$$

(8b) $$\begin{array}{cc} \; & X_{w}\geq Y_{wu},\;w\in V,u\in \delta^{+}\left(w\right) \end{array}$$

(8c) $$\begin{array}{cc} \; & X_{w}\leq \sum_{u\in \delta^{+}\left(w\right)}Y_{wu},\;w\in V \end{array}$$

(8d) $$\begin{array}{cc} \; & X_{w}+\sum_{u\in \delta^{-}\left(w\right)}Y_{uw}\leq 1,\;w\in V \end{array}$$

(8e) $$\begin{array}{cc} \; & \sum_{m\in M}z_{w}^{m}=X_{w},\;w\in V \end{array}$$

(8f) $$\begin{array}{cc} \; & \sum_{p\in P}h_{w}^{p}=X_{w},\;w\in V \end{array}$$

(8g) $$\begin{array}{cc} \; & \sum_{p\in P}h_{w}^{p}P\left(p\right)G\left(w,u\right)+M\left(w,u,m\right)\left(1-Y_{wu}\right)\geq \\ \; & \gamma \left(m\right)z_{w}^{m}\eta +\gamma \left(m\right)\sum_{v\in V\setminus \{w,u\}}\sum_{p\in P}P\left(p\right)G\left(v,u\right)Z_{wv}^{mp},\;\left(w,u\right)\in A,m\in M \end{array}$$

(8h) $$\begin{array}{cc} \; & Z_{wv}^{mp}\leq z_{w}^{m},\;Z_{wv}^{mp}\leq h_{v}^{p},\;Z_{wv}^{mp}\geq h_{v}^{p}+z_{w}^{m}-1,\;m\in M,w\in V,v\in V\setminus \left\{w\right\},p\in P \end{array}$$

(8i) $$\begin{array}{cc} \; & U_{swv}\leq g_{sw},\;U_{swv}\leq Y_{wv},\;s\in S,w\in V,v\in \delta^{+}\left(v\right) \end{array}$$

(8j) $$\begin{array}{cc} \; & \sum_{s\in S}g_{sw}\leq \sum_{m\in M}B\left(m\right)F_{w}^{m},\;w\in V;\;\;g_{sw}\leq 1,\;s\in S,w\in V \end{array}$$

(8k) $$\begin{array}{cc} \; & F_{w}^{m}\leq \left|S\right|z_{w}^{m},\;F_{w}^{m}\leq f,F_{w}^{m}\geq f-\left|S\right|\left(1-z_{w}^{m}\right),\;m\in M,w\in V \end{array}$$

(8l) $$\begin{array}{cc} \; & Y_{wv}\in B,\;w\in V,v\in \delta^{+}\left(w\right);\;X_{w}\in B,\;w\in V \end{array}$$

(8m) $$\begin{array}{cc} \; & z_{w}^{m}\in B,\;m\in M,w\in V;\;h_{v}^{p}\in B,\;p\in P,v\in V \end{array}$$

(8n) $$\begin{array}{cc} \; & f\in R;\;g_{sw}\in R_{+},\;s\in S,w\in V\;F_{w}^{m}\in R,\;m\in M,w\in V \end{array}$$

(8o) $$\begin{array}{cc} \; & Z_{wv}^{mp}\in B,\;m\in M,p\in P,w\in V,v\in V\setminus \left\{w\right\};\;U_{swv}\in R_{+},\;s\in S,w\in V,v\in \delta^{+}\left(w\right). \end{array}$$

As compared with formulation (7), now a new set of binary variables is used, namely $h_{w}^{p},p\in P,w\in V$, where $h_{w}^{p}$ equals 1 if, and only if, node *w* transmits with the power level *p*, and 0 otherwise. Subsquently, new constraint (8f) is introduced to ensure that each transmitting node is assigned exactly one transmitting power level from P. Finally, the SINR constraint is modified in the following way: p(w,v) is replaced by multiplying the power P(p) (corresponding to the power level *p* assigned to node *w* that is identified by variable $h_{w}^{p}$) by the path gain denoted by G(w,v). Finally, binary variables $Z_{wv}^{mp}\in B,\;m\in M,p\in P,w\in V,v\in V\setminus \left\{w\right\}$, together with additional constraints (8h), are introduced to eliminate bi-linearities that otherwise occur because of the multiplication $h_{v}^{p}z_{w}^{m}$.

### 6.3. Continuous Power Control

Contrary to the discrete power control case, in the continuous power control the transmitting power that is applied by the nodes can change continuously. To express this, PP is modified in the following way:

(9a) $$\begin{array}{cc} \; & max\;\;P=-f+\sum_{s\in S}\sum_{w\in V}\sum_{v\in \delta^{+}\left(w\right)}\lambda_{s(w,v)}^{*}U_{swv} \end{array}$$

(9b) $$\begin{array}{cc} \; & X_{w}\geq Y_{wu},\;w\in V,u\in \delta^{+}\left(w\right) \end{array}$$

(9c) $$\begin{array}{cc} \; & X_{w}\leq \sum_{u\in \delta^{+}\left(w\right)}Y_{wu},\;w\in V \end{array}$$

(9d) $$\begin{array}{cc} \; & X_{w}+\sum_{u\in \delta^{-}\left(w\right)}Y_{uw}\leq 1,\;w\in V \end{array}$$

(9e) $$\begin{array}{cc} \; & \sum_{m\in M}z_{w}^{m}=X_{w},\;w\in V \end{array}$$

(9f) $$\begin{array}{cc} \; & p_{w}G\left(w,u\right)+M\left(w,u,m\right)\left(1-Y_{wu}\right)\geq \\ \; & \;\gamma \left(m\right)z_{w}^{m}\eta +\gamma \left(m\right)\sum_{v\in V\setminus \{w,u\}}G\left(v,u\right)P_{wv}^{m},\;\left(w,u\right)\in A,m\in M \end{array}$$

(9g) $$\begin{array}{cc} \; & P_{min}X_{w}\leq p_{w}\leq P_{max}X_{w},\;w\in V \end{array}$$

(9h) $$\begin{array}{cc} \; & P_{wv}^{m}\leq P_{max}z_{w}^{m},\;m\in M,w\in V,v\in V\setminus \left\{w\right\} \end{array}$$

(9i) $$\begin{array}{cc} \; & P_{wv}^{m}\leq p_{v},\;m\in M,w\in V,v\in V\setminus \left\{w\right\} \end{array}$$

(9j) $$\begin{array}{cc} \; & P_{wv}^{m}\geq p_{v}-\left(1-z_{w}^{m}\right)P_{max},\;m\in M,w\in V,v\in V\setminus \left\{w\right\} \end{array}$$

(9k) $$\begin{array}{cc} \; & U_{swv}\leq g_{sw},\;U_{swv}\leq Y_{wv},\;s\in S,w\in V,v\in \delta^{+}\left(v\right) \end{array}$$

(9l) $$\begin{array}{cc} \; & \sum_{s\in S}g_{sw}\leq \sum_{m\in M}B\left(m\right)F_{w}^{m},\;w\in V;\;\;g_{sw}\leq 1,\;s\in S,w\in V \end{array}$$

(9m) $$\begin{array}{cc} \; & F_{w}^{m}\leq \left|S\right|z_{w}^{m},\;F_{w}^{m}\leq f,F_{w}^{m}\geq f-\left|S\right|\left(1-z_{w}^{m}\right),\;m\in M,w\in V \end{array}$$

(9n) $$\begin{array}{cc} \; & Y_{wv}\in B,\;w\in V,v\in \delta^{+}\left(w\right);\;X_{w}\in B,\;w\in V \end{array}$$

(9o) $$\begin{array}{cc} \; & z_{w}^{m}\in B,\;m\in M,w\in V;\;p_{w}\in R_{+},\;w\in V;\;P_{wv}^{m}\in R_{+},\;m\in M,w\in V,v\in V\setminus \left\{w\right\} \end{array}$$

(9p) $$\begin{array}{cc} \; & f\in R;\;g_{sw}\in R_{+},\;s\in S,w\in V;\;F_{w}^{m}\in R,\;m\in M,w\in V \end{array}$$

(9q) $$\begin{array}{c} U_{swv}\in R_{+},\;s\in S,w\in V,v\in \delta^{+}\left(w\right). \end{array}$$

Instead of binary variables $h_{w}^{p}$ used in the previous formulation, the continuous variables $p_{w},w\in V$ are now introduced to express the actual power that is applied at node *w*. These variables are used in the SINR constraint modified analogously to the discrete case. It is assumed that the value of $p_{w}$ cannot be smaller than the minimum power $P_{min}$ (a parameter), and it cannot exceed the maximum power $P_{max}$—this is assured by the constraints (9g). Variables $P_{wv}^{m}$, together with constraints (9h)–(9j), are the auxiliary constraints used to get rid of bi-linearities that otherwise appear in the SINR constraint because of the multiplication $z_{w}^{m}p_{v}$.

## 7. Numerical Study

Below, we present the numerical results that were obtained by means of the optimization approach described in the previous sections. We first consider the primary aim of the study, i.e., answering the question to what extent network traffic throughput can be increased through joint MCSs assignment and TPC. For that, discrete and continuous power control are both considered in the study. Later, on top of that, we discuss the computation time efficiency of the considered optimization approach.

All of the optimization problems considered in the paper were implemented and solved by means of C# and CPLEX 12.9.0, respectively. All of the computations were executed on an Intel Core i7-8550U CPU (four cores, each up to 4 GHz) with 16 GB RAM.

### 7.1. Network Setting

**Figure 1:**
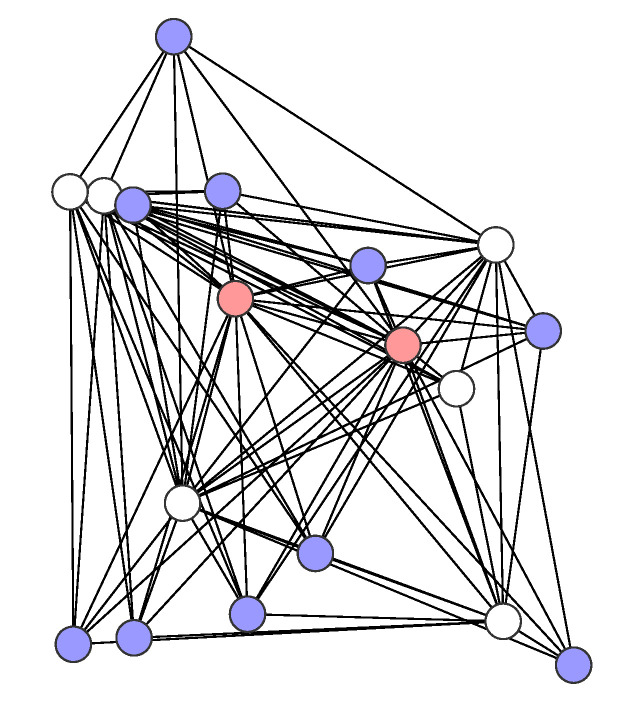
A small network example.

**Figure 2:**
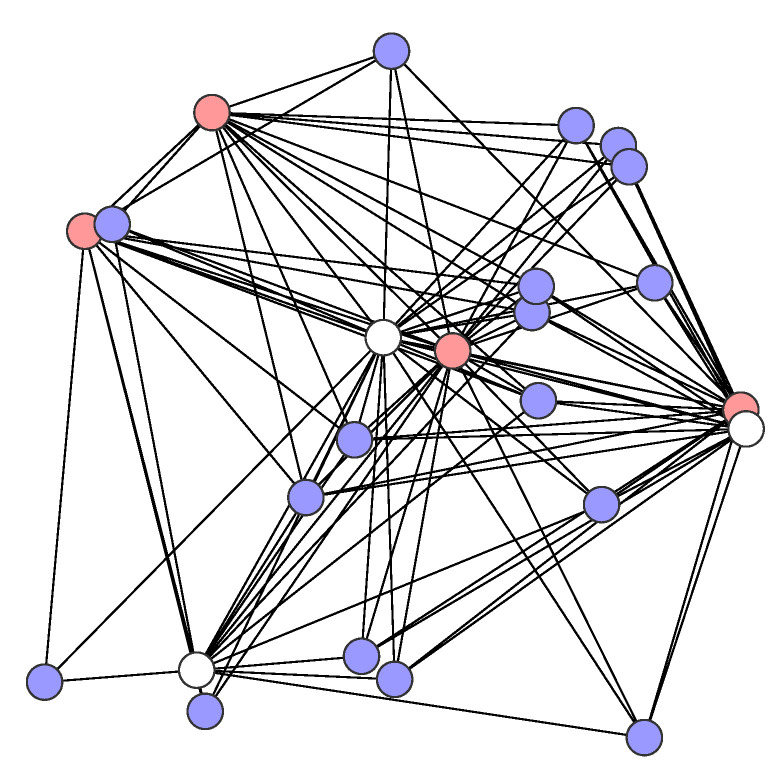
A medium network example.

**Figure 3:**
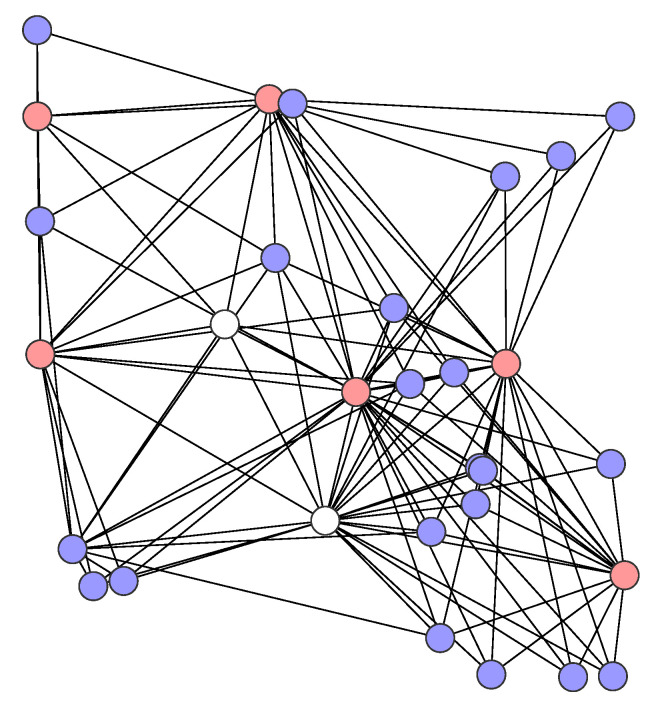
A large network example.

In the study, we consider irregular networks that were generated by [38] of three different sizes: small networks with 18 nodes, medium networks with 24 nodes, and large networks with 30 nodes. In each network, the nodes were placed in a given square area according to the uniform distribution. The size of the network area is equal to 220 m × 220 m, 250 m × 250 m, and 280 m × 280 m, respectively, for small, medium, and large networks. Each small networks has two sensors and 10 destinations, each medium network has four sensors and 16 destinations, while each large network has six sensors and 22 destinations; all of the remaining nodes in each network serve as purely transit nodes. Figure 1, Figure 2 and Figure 3 show the example networks. The sensor nodes are depicted in red, destinations in blue, and transit nodes in white.

In the study, we assume that the noise power η is equal to −101 dBm and that the network nodes are operating in 5 GHz band. The power that is received at the nodes is calculated by means of the simplified path-loss model from [39]:

(10) $$p\left(w,u\right)=P\cdot G\left(w,u\right)=P{\left(\frac{\lambda }{4\pi d_{0}}\right)}^{2}{\left(\frac{d_{0}}{d}\right)}^{\alpha },$$

where p(w,u) (as expressed in mW) is the power that is received by node *u* when node *w* is broadcasting, *P* is the transmission power of node *w*, $d_{0}$ (in m) is a reference distance, *d* (in m) is the distance between *w* and *u*, λ (in m) is the signal wavelength, and α is the path-loss exponent. In the study, we assume $d_{0}=$ 10 m and α=4. As mentioned above, the transceivers are operating in 5 GHz band and, thus, λ= 0.06 m.

Clearly, the maximum transmission range depends on the transmission power and the MCS used by the broadcasting node. In the following, we assume that the set of available MCSs M is composed of three MCSs: BPSK 3/4, 16-QAM 1/2, and 16-QAM 3/4. The power ratio thresholds γ(m) and transmission rates B(m) of these MCSs are, respectively, equal to 6.5 dB and 12 Mbps, 12.8 dB and 18 Mbps, and 16.2 dB and 24 Mbps [34]. The maximum transmission range equals 170 m for the assumed MCS and the maximum transmission power considered in this study that is equal to 130 mW.

### 7.2. Joint Modulation and Coding Schemes Assignment and Transmission Power Control

In this section, we are going to answer the main research question that is posed in this article. For the generated network instances, we analyze to what extent traffic throughput can be increased by joint MCSs assignment and TPC. Because the impact of the MCSs assignment is comprehensively discussed in [17], in this study we skip the analysis of all possible MCSs subsets combinations and focus on the following four cases differing in the number of MCSs available for each node and the type of TPC applied:

- A—one MCS from the set of available MCSs M, namely BPSK 3/4, and no TPC; the transmission power of each node equals 90 mW,
- B—all three MCSs from the set of available MCSs M and no TPC; the transmission power of each node equals 90 mW,
- C—all three MCSs from the set of available MCSs M and discrete TPC; the transmission power of each node can take the value from set {50 mW, 90 mW, 130 mW},
- D—all three MCSs from the set of available MCSs M and continuous TPC; the transmission power of each node can take the values from range [50 mW, 130 mW].

**Figure 4:**
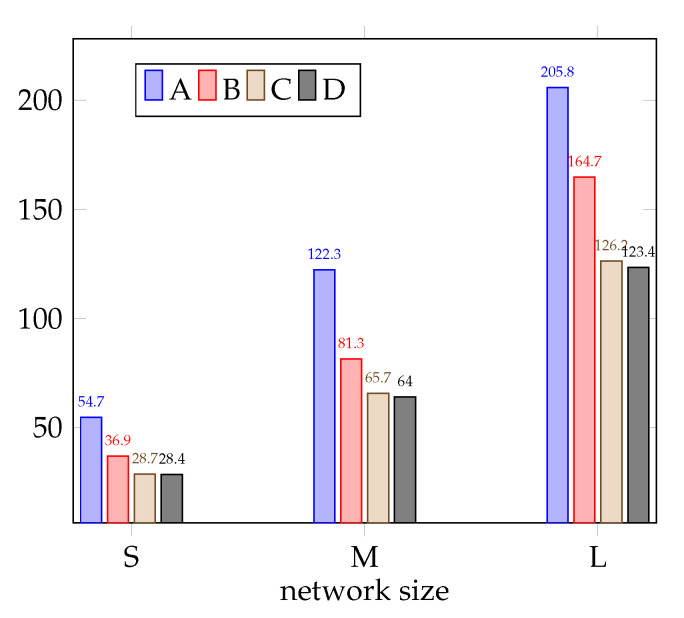
Frame size as a function of the network size.

The results, i.e., the frame sizes, which were obtained for the considered cases are presented in Table 2, Table 3 and Table 4, respectively, for small, medium, and large networks. Each table contains results for ten randomly generated networks of a given size as well as the results that are averaged over all ten network instances. All of the frame sizes reported in this section are expressed in the number of time slots. The averaged results are then illustrated in Figure 4.

The presented results show that the significant gain can be achieved by applying all three available MCSs. As far as the averaged results are concerned, the value of this gain is equal to 32.5%, 33.5%, and 19.9% for small, medium, and large networks, respectively. The frame size can be then significantly decreased by applying TPC; however, it is not the most essential whether it is discrete or continuous TPC. For small networks, the average frame size after applying discrete TPC is further decreased by 22.2%, and the difference between discrete TPC and continuous TPC only equals 1%. Note that, in nine out of ten small networks, it does not matter which type of TPC we choose—a difference only exists for Network 7. The difference is also not very significant when it comes to the medium networks. When compared to case B, the averaged frame size can be decreased by 19.2% by applying discrete TPC and by 21.3% applying continuous. However, contrary to the previous case, small differences between discrete TPC and continuous TPC exist for most networks. The similar, again not very significant, difference between discrete and continuous TPC is for large networks. In this case, the averaged frame size is decreased by 23.1% by applying discrete TPC and by 25.1% when continuous TPC is used. Note that these values applied to the averaged results; however, it should be noticed that the influence of the TPC strongly depends on the network topology (compare, for example, Network 8 and Network 9). Finally, we should highlight the substantial different between case A (no power control with single MCS) and case D (continuous power with multiple MCSs), which is equal to 48.1%, 47.7%, and 40.1% for small, medium, and large networks when it comes to the averaged results.

### 7.3. Time Efficiency of the Optimization Model

In this section, we analyze computation times of the solution algorithm applied in our optimization model based on the CPLEX solver. Table 5 summarizes the notations that are used in this section.

Table 6, Table 7 and Table 8 present the averaged results illustrating time efficiency of the optimization model for small, medium, and large networks. The meaning of the letters A, B, C, and D in the columns’ headers is the same as in the previous section. Additionally, the unit of the frame size remains the same, i.e., all of the frame sizes are expressed in the number of time slots (note that, in the case of the linear relaxation of the problem, the number of time slots does not have to be integer).

We first note that each problem variant, even for large networks, was solved in a reasonable time. However, the time that is needed to solve the problem increases significantly with the network size. Second, including MCSs assignment and TPC leads to a substantial increase of the number of generated c-sets. This suggests that it is the increased number of simultaneous transmissions possible that lead to the gain in traffic throughput discussed in the previous section. Finally, the quality of linear relaxation is not too high (contrary to the basic optimization model from [14]). This issue should be addressed in the future work, since it affects the effectiveness of the branch-and-bound (B&B) algorithm underlying the CPLEX solver.

## 8. Remarks

There are several aspects that are out of the scope of this paper that we believe the reader should know about. All of these aspects will be discussed in this section.

First, although three different network sizes have been considered in this paper, it is important to also analyze different networks topologies. In fact, our model is topology-agnostic in the sense that it works for general directed graph structure. However, for the particular topologies that are encountered in such networks as scale-free networks or small-world networks that possess certain specific properties (see [40]), it would be interesting to analyze these kind of networks in order to take advantage of the properties of their topologies to improve the general solution algorithm proposed in this paper.

Second, as previously stated, our aim is to provide a rigid exact mathematical model for the considered problem (recall that the problem is NP-hard). This assumption is not without its consequences. Namely, in general, we cannot expect to solve large problem instances (of, say, hundreds or more nodes) in an acceptable time since the model is developed within the integer-programming optimization framework that uses B&B as the basic solution approach. In such a case, appropriate heuristics should be considered.

Finally, we would like to give some additional remark on the time efficiency of the proposed solution algorithm. As the reader may have noticed, the solution time is mainly determined by the time that is needed to solve the MIP problems PP and FMP, and this time grows significantly with the network size. Nevertheless, despite the algorithm complexity, the proposed medium sized examples were solved in a reasonable time.

## 9. Conclusions

In the paper, we have presented an extension of the optimization model introduced in [14]. The extension makes it possible to deal with the complex problem of including transmission power control into throughput optimization in WSNs with dynamic MCSs assignment that serve multicast traffic. The numerical results that were obtained by implementing the optimization model show that adding TPC on top of dynamic MCSs assignment leads to a significant gain in traffic throughput. This gain is considerable for both discrete and continuous TPC, and the difference between those two cases is usually negligible. The main appplication scenario of the introduced optimization model is to use it as a benchmark while designing new algorithms and protocols that are intended for WSNs. As far as the ongoing and future work is concerned, we are extending our model by introducing multi-criteria optimization in order to jointly minimize the frame size and energy consumption. Finally, the quality of the linear relaxation needs to be addressed. All of these issues are already under consideration within the grant mentioned in the acknowledgement.

## Figures and Tables

**Table 1:** Notation: network setting.

Notation	Description
V	set of nodes
S,D,Q	sensors, destinations, transit nodes, respectively
A	set of directed radio links (arcs); a=(b(a),e(a))
$\delta^{+}\left(v\right)$	set of nodes connected with *v* by its outgoing arcs
$\delta^{-}\left(v\right)$	set of nodes connected with *v* by its incoming arcs
p(v,w)	power received at node *w* from transmitting node *v*
η	noise power
*m*	modulation and coding scheme
M	set of MCSs (M:={1,2,…,M})
γ(m)	SINR threshold for MCS *m*
B(m)	bitrate in (Mbps) for MCS *m*
*c*	compatible set (c-set in short)
m(c,w)	number of the MCS assigned to node *w* in c-set *c*
$\hat{C}$	family of all c-sets
W(c)	set of nodes transmitting when c-set *c* is used
U(c,w)	set of nodes that receive signal from w∈W(c)
C	selected subfamily of $\hat{C}$
C(a)	subset of c-sets in C broadcasting over arc a C(a)=C(b(a),e(a))
D(s)	set of destination nodes of sensor *s* (D(s)⊆D)
B(s)	multicast routing tree rooted at *s* with leaves D(s)
V(s),A(s)	set of nodes and arcs, respectively, of B(s)
$B,Z,R_{+}$	B={0,1}, Z integers, $R_{+}$ non-negative real numbers

**Table 2:** Optimized frame sizes for small networks

	A	B	C	D
Network 1	54	35	31	31
Network 2	41	19	14	14
Network 3	41	31	32	32
Network 4	68	50	33	33
Network 5	68	43	31	31
Network 6	95	70	41	41
Network 7	41	25	23	20
Network 8	70	43	36	36
Network 9	28	21	14	14
Network 10	41	32	32	32
Avg	54.7	36.9	28.7	28.4

**Table 3:** Optimized frame sizes for medium networks.

	A	B	C	D
Network 1	110	84	79	78
Network 2	97	84	63	63
Network 3	109	64	52	51
Network 4	176	132	98	97
Network 5	121	64	51	50
Network 6	95	49	44	44
Network 7	110	62	52	50
Network 8	148	100	63	61
Network 9	122	91	95	88
Network 10	135	83	60	58
Avg	122.3	81.3	65.7	64

**Table 4:** Optimized frame sizes for large networks.

	A	B	C	D
Network 1	162	133	118	116
Network 2	242	194	122	121
Network 3	149	107	108	107
Network 4	188	141	124	119
Network 5	215	194	125	121
Network 6	202	126	114	111
Network 7	190	137	125	121
Network 8	254	218	144	144
Network 9	175	239	105	101
Network 10	281	258	177	173
Avg	205.8	164.7	126.2	123.4

**Table 5:** Numerical results—explanation.

Notation	Description
$T^{LR}$	optimal frame size obtained from the linear relaxation of the problem after the c-sets generation process
$T^{MIP}$	optimal frame size obtained from frame size minimization
|C|	number of generated c-sets
$t^{MP}$	total computation time of solving master problem during c-sets generation process
$t^{PP}$	total computation time of solving pricing problem during c-sets generation process
$t^{MIP}$	computation time of solving the final MIP version of the problem

**Table 6:** Optimization model efficiency-small networks.

	A	B	C	D
$T^{LR}$	42.4	30.2	21.2	21.1
$T^{MIP}$	54.7	36.9	28.7	28.4
|C|	13.4	24.7	42.2	41.3
$t^{MP}$	1.6 s	2.9 s	6.2 s	6.1 s
$t^{PP}$	4.5 s	51.1 s	9 m 49 s	2 m 48 s
$t^{MIP}$	0.4 s	0.5 s	0.6 s	0.7 s

**Table 7:** Optimization model efficiency-medium networks.

	A	B	C	D
$T^{LR}$	91.09	65.89	50.50	49.86
$T^{MIP}$	122.3	81.3	65.7	64
|C|	28.4	62.2	61.6	71.3
$t^{MP}$	22.4 s	47.7 s	563s	1 m 6 s
$t^{PP}$	30.2 s	6 m 56 s	16 m 57 s	14 m 44 s
$t^{MIP}$	4 m 21 s	3 m 37 s	1 m 46 s	4 m 44 s

**Table 8:** Optimization model efficiency-large networks.

	A	B	C	D
$T^{LR}$	167.92	140.82	101.43	100.32
$T^{MIP}$	205.8	164.7	126.2	123.4
|C|	41.5	79.8	86	103.7
$t^{MP}$	1 m 7 s	2 m 17 s	2 m 52 s	3 m 35 s
$t^{PP}$	41 s	12 m 21 s	23 m 51 s	25 m 15 s
$t^{MIP}$	12 m 42 s	4 m 44 s	12 m 47 s	13 m 47 s

## Data Availability

Not applicable.
